# Cost-effectiveness of cardiac rehabilitation: a systematic review

**DOI:** 10.1136/heartjnl-2017-312809

**Published:** 2018-04-13

**Authors:** Gemma E Shields, Adrian Wells, Patrick Doherty, Anthony Heagerty, Deborah Buck, Linda M Davies

**Affiliations:** 1 Centre for Health Economics, University of Manchester, Manchester, UK; 2 School of Psychological Sciences, University of Manchester, Manchester, UK; 3 Manchester Mental Health and Social Care NHS Trust, Manchester, UK; 4 Department of Health Sciences, University of York, York, UK; 5 Institute of Cardiovascular Sciences, University of Manchester, Manchester, UK

**Keywords:** cardiac rehabilitation, health care economics, systemic review

## Abstract

Patients may be offered cardiac rehabilitation (CR), a supervised programme often including exercises, education and psychological care, following a cardiac event, with the aim of reducing morbidity and mortality. Cost-constrained healthcare systems require information about the best use of budget and resources to maximise patient benefit. We aimed to systematically review and critically appraise economic studies of CR and its components. In January 2016, validated electronic searches of the National Health Service Economic Evaluation Database (NHS EED), Health Technology Assessment, PsycINFO, MEDLINE and Embase databases were run to identify full economic evaluations published since 2001. Two levels of screening were used and explicit inclusion criteria were applied. Prespecified data extraction and critical appraisal were performed using the NHS EED handbook and Drummond checklist. The majority of studies concluded that CR was cost-effective versus no CR (incremental cost-effectiveness ratios (ICERs) ranged from $1065 to $71 755 per quality-adjusted life-year (QALY)). Evidence for specific interventions within CR was varied; psychological intervention ranged from dominant (cost saving and more effective) to $226 128 per QALY, telehealth ranged from dominant to $588 734 per QALY and while exercise was cost-effective across all relevant studies, results were subject to uncertainty. Key drivers of cost-effectiveness were risk of subsequent events and hospitalisation, hospitalisation and intervention costs, and utilities. This systematic review of studies evaluates the cost-effectiveness of CR in the modern era, providing a fresh evidence base for policy-makers. Evidence suggests that CR is cost-effective, especially with exercise as a component. However, research is needed to determine the most cost-effective design of CR.

## Introduction

Globally, the prevalence of cardiovascular disease is increasing due to ageing and population growth.^1^ Following a cardiac event, patients may be offered cardiac rehabilitation (CR), a supervised programme, typically including exercises, health education and psychological intervention.^2^ Evidence suggests that CR programmes can reduce morbidity and mortality following a cardiac event, along with increasing quality of life and psychological well-being.^3^

In the UK, approximately 88 000 people start CR annually.^4^ The average cost is reported at £477 per person (2010, UK pounds sterling), representing a potential total cost of around £42 million annually.^5^ CR programmes have been shown to reduce unplanned hospital admissions, with the potential to save health systems resources and reduce the burden on already stretched cardiac departments.^3^

Where there is a growing demand placed on the healthcare system but with limited budgets, economic evaluation supports decision-making. Different types of economic evaluations can be conducted. Cost-effectiveness analysis (CEA) measures the cost and the clinical impact (health benefit) of an intervention and translates into a single value: the incremental cost-effectiveness ratio (ICER). Cost–utility analysis falls within the CEA type of economic evaluation but uses utilities and life expectancy to show differences in health benefit. Cost–benefit analysis expresses outcomes in monetary units. Finally, cost-minimisation analysis is only appropriate if there is clear evidence that interventions are of equal health benefit.

Two earlier reviews of economic evaluations for interventions in CR^6 7^ found evidence to support the cost-effectiveness of CR intervention. However, evidence was limited by study quality, variation across CR design and delivery, and uncertainty. More recently, a review focused on economic evaluations of CR interventions in low/middle-income countries.^8^ This identified that CR intervention was cost-effective in heart failure patients, although, intervention cost was a key issue. Our study adds a fresh perspective by focusing on full economic evaluations (synthesising costs and health benefits) to allow for a truer assessment of cost-effectiveness and updates the literature as developments in cardiac treatment mean that older literature is no longer relevant.^2 9^

The current review aimed to answer the following question: is CR cost-effective in the modern era (post stent and with statins), compared to alternatives or no intervention? Our secondary research question was to determine the effects of modes of delivery and core components of CR on the cost-effectiveness of CR in the modern era. We also critically appraise the evidence to identify data gaps and inform future research needs.

## Methods

A systematic literature search and review was conducted to identify economic evaluations of CR interventions. The protocol was registered on the PROSPERO register of systematic reviews (CRD42016050725)

### Searches

An electronic literature search was conducted in January 2016 (updated January 2017) using the PsycInfo, MEDLINE, Embase, National Health Service Economic Evaluation Database (NHS EED) and Health Technology Assessment databases.

Searches were structured to identify cost-effectiveness evidence, published in English since 2001. Due to developments in therapies, surgery and medications offered in CR in recent years, older studies are no longer relevant, hence restriction is justified.^9 10^ Common search terms included CR terms and economic evaluation terms. Search terms for economic evaluations were taken from the Centre for Reviews and Dissemination.^11^ Intervention terms were taken from previously published search strategies.^12 13^ Medical subject headings were combined with free-text terms to form search strategies. Terms varied according to database designs. Search terms are provided in the online supplementary material.

10.1136/heartjnl-2017-312809.supp1 Supplementary file 1

### Selection

Following database searching, titles and abstracts of identified citations were manually screened to assess their relevance to the review. Explicit inclusion criteria were as follows: (1) studies focusing on adults offered CR in line with National Institute for Health and Care Excellence eligibility guidelines^14–16^; (2) CR programmes or specific interventions within CR were eligible as interventions; (3) alternative interventions and usual care were accepted comparators; (4) studies had to be primary studies including a full economic evaluation, comparing interventions in terms of cost-effectiveness, cost–utility, cost–benefit or cost minimisation analysis.

Following the first round of screening, full text articles were obtained and were reassessed against the eligibility criteria. Two reviewers (GES and DB) carried out each screening stage independently; differences in opinion were discussed and decided with a third reviewer (LMD).

### Data extraction and synthesis

Data extraction included study objectives, methods and results. Studies were critically appraised using data extracted consistent with the NHS EED handbook and Drummond checklist.^17 18^

Data extraction was performed by two reviewers (GES and DB), with results cross-checked and discussed and finalised with the assistance of a third reviewer (LMD). Due to the heterogeneity among studies, a narrative synthesis, rather than a quantitative synthesis, was used to summarise findings.

Cost data were converted to 2016 US$, using the consumer price index and purchasing power parity conversion factor, to allow for easier comparison between studies.^19 20^

## Results

**Figure 1:**
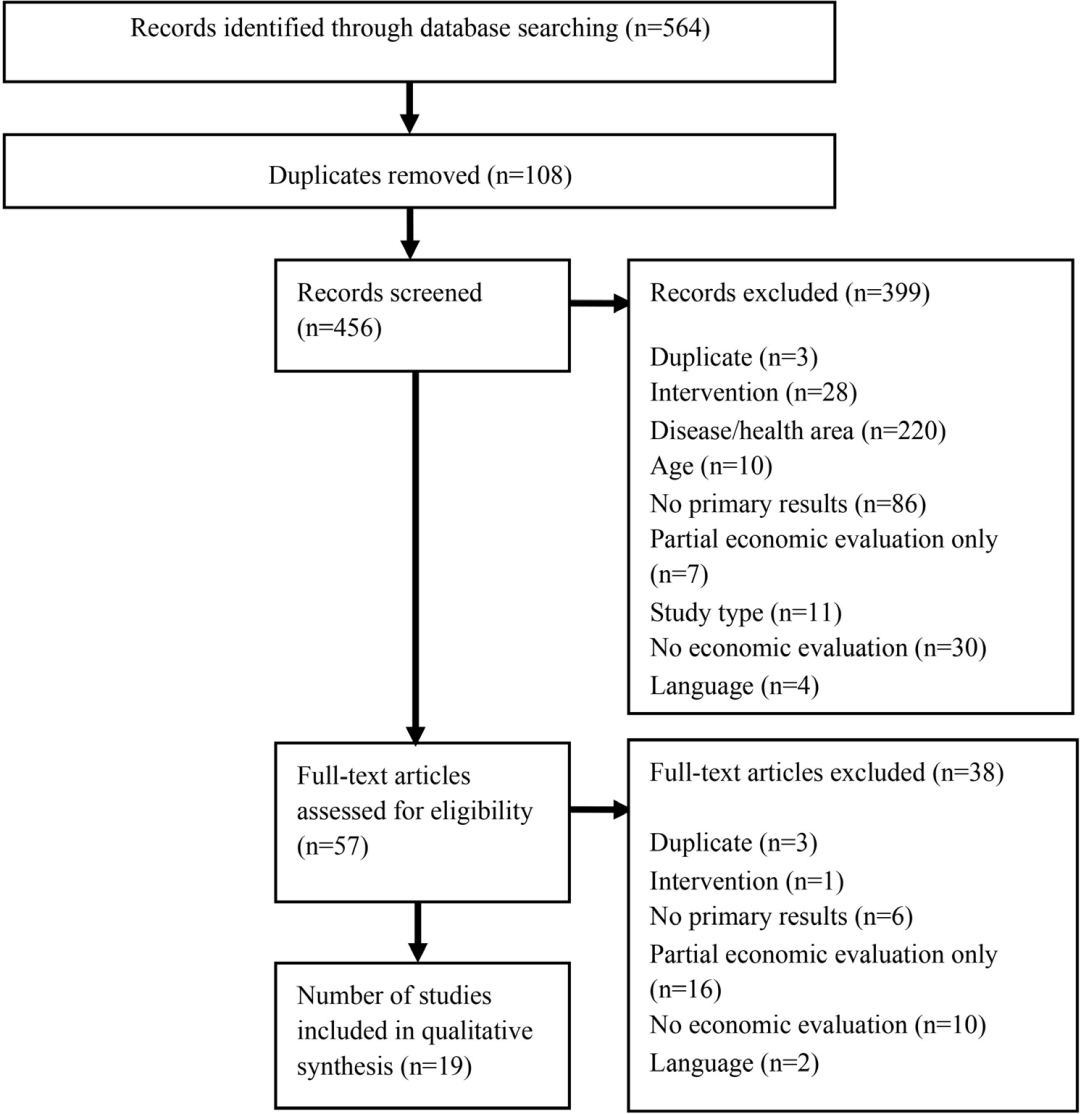
Flow diagram of search results.

There were 564 initial search results; following screening of titles/abstracts, 57 articles were assessed. Nineteen studies were included in the review (figure 1).

An overview of study characteristics is given in table 1.

**Table 1:** Study overview

Study	Population	Setting	Intervention	Comparator	Outcomes	Time horizon
Comparing CR with no CR						
Georgiou *et al*, 2001^21^	Stable chronic heart failure	Outpatient care in the USA	Long-term moderate exercise training	No exercise training	LYG	15.5 years
Briffa *et al*, 2005^30^	Patients who had had an uncomplicated acute myocardial infarction or recovered from unstable angina	Tertiary hospital care in Australia	Comprehensive CR plus usual care	No CR	QALYs	12 months
Huang *et al*, 2008^22^	Patients with end-stage renal disease who initiated chronic haemodialysis and underwent CABG	Outpatient care in USA	CR	No CR	LYG	4 years
Oldridge *et al*, 2008^23^	Myocardial infarction with anxiety and depression	Outpatient care in the USA	CR	No CR	QALYs	12 months
Leggett *et al*, 2015^34^	Patients undergoing a cardiac catheterisation for myocardial infarction or stable or unstable angina	Outpatient care in Canada	Centre-based outpatient CR programme	No CR	QALYs	Lifetime
Rincón *et al*, 2016^35^	Chronic heart failure	Outpatient care in Columbia	Exercise-based CR plus UC	UC (no CR programme)	QALYs and LYG	5 years
De Gruyter *et al*, 2016^36^	Myocardial infarction	Outpatient care in Australia	CR (uptake of 50% and 65%)	CR (uptake of 30%)	DALYs	10 years
Comparing exercise components of CR with education						
Yu *et al*, 2004^31^	Patients with recent myocardial infarction or percutaneous coronary intervention	Outpatient care in Hong Kong	CR and prevention programme (exercise and education)	UC (education only)	QALYs	2 years
Reed *et al*, 2010^24^	Medically stable outpatients with heart failure and reduced ejection fraction	Outpatient care in the USA	Exercise training plus UC	UC (education only)	QALYs	2.5 years
Kühr *et al*, 2011^37^	Clinically stable heart failure	Outpatient care in Brazil	Supervised exercise therapy alongside standard care	Standard care	QALYs and LYG	10 years
Comparing telehealth interventions with CR based in a healthcare centre						
Cheng *et al*, 2016^38^	Patients with cardiac disease who were referred to but did not attend a rehabilitation programme	Home-based care in Australia	Four pedometer-based telephone coaching sessions on weight, nutrition and physical activity	Two pedometer-based telephone coaching sessions on physical activity alone or information only	QALYs	30 years
Maddison *et al*, 2015^25^	Ischaemic heart disease	Community care in New Zealand	Heart exercise and remote technologies mobile phone intervention plus UC	UC (exercise and cardiac support group)	QALYs	24 weeks
Frederix *et al*, 2016^32^	Coronary artery disease, percutaneous coronary intervention or with CABG or chronic heart failure	Outpatient care in Belgium	Cardiac telerehabilitation programme in addition to conventional centre-based CR	Centre-based CR programme	QALYs	24 weeks
Kidholm *et al*, 2016^26^	Artery sclerosis, CABG, valve surgery or heart failure	Outpatient care in Denmark	ICT delivered individualised cardiac telerehabilitation programme	Traditional rehabilitation programme at the hospital or healthcare centre	QALYs	12 months
Comparing distribution of CR programmes						
Papadakis *et al*, 2008^33^	Coronary artery disease	Outpatient care in Canada	CR programme distributed over 12 months	Standard CR over 3 months	QALYs	24 months
Comparing care settings of CR programmes						
Taylor *et al*, 2007^27^	Uncomplicated acute myocardial infarction (without major comorbidity)	Home-based or outpatient care in the UK	Home-based CR	Hospital-based rehabilitation	QALYs	9 months
Schweikert *et al*, 2009^28^	Patients with an acute coronary event such as ST-elevation myocardial infarction, non-STEMI or unstable angina	Inpatient or outpatient care in Germany	Outpatient CR	Inpatient CR	QALYs	12 months
Comparing psychological intervention with UC						
Lewin *et al*, 2009^40^	Heart disease patients undergoing implantation of a cardiac defibrillator	Outpatient care in the UK	Home-based cognitive–behavioural programme	UC (information booklet)	QALYs	6 months
Dehbarez *et al*, 2015^29^	Ischaemic heart disease and heart failure	Outpatient care in Denmark	Learning and coping education strategies	UC (standard CR)	QALYs	5 months

CR, cardiac rehabilitation; CABG, coronary artery bypass grafting; DALYs, disability adjusted life-years; ICT, information and communication technology; LYG, life-years gained; QALYs, quality-adjusted life-years; STEMI, ST-elevation myocardial infarction; UC, usual care.

### Critical appraisal

As expected, table 1 shows wide variation in study populations because CR is recommended for multiple patient groups.^14^ Variation between studies and the lack of a pool of studies with the same well-defined population characteristics means that we cannot clearly differentiate between population groups; instead we consider the group as a whole. This review focuses on a cardiac population in the modern era (post stent and with statins); the use of stents or statins was not reported in nine of the studies.^21–29^ Reported use of statins varied from 46% to 98%, and the proportion of the population admitted to CR following a stent ranged greatly (8%–82%).^30–33^

### Interventions

Seven studies compared CR with no CR programme^21–23 30 34–36^; remaining studies compared intervention types within CR (see table 1). Publication dates suggest that more recent interest in CR has been around the role of telehealth and how frequent monitoring within CR, aided by information and communication technologies (ICTs), can make an impact.

The design of CR varied greatly within the studies. Three studies did not describe the content of CR.^22 34 36^ The most common intervention included was exercise/physical activity (14/19).^21 23 25–28 30–33 35 37–39^ Seven studies included education and information.^26–28 30–33^ Of these, three studies did not describe the intervention.^27 28 33^ The remainder reported limited information, one was aimed at symptom management and healthy eating, two focused on risk factor modification and one provided information on rehabilitation topics.^26 30–32^ Psychological intervention was the least common component included (6/19), ranging from stress management tips to more-intensive psychosocial counselling.^23 27–30 40^ Two studies focused on care settings.^27 28^ One study compared hospital-based (outpatient) CR with home-based (manual step-by-step guide) CR, components of CR were the same.^27^ There were some key differences; outpatient rehabilitation was delivered by a multidisciplinary team, whereas home-based patients communicated only with a CR nurse and home-based CR initiated sooner after discharge.^27^ In the second study, the content of CR remained the same with inpatient and outpatient settings compared; both arms were subjected to identical CR for 6 hours per day.^28^ Telehealth intervention design varied greatly. Three studies measured physical activity using devices: one with a pedometer-based intervention and telephone coaching,^38^ one with an accelerator and personalised training protocols^32^ and one with a comprehensive package of devices to measure activity and to monitor health which was shared with healthcare providers.^26^ A further study simply delivered a personalised, automated package of text messages and provided details to a website with further information.^25^

The most common comparator was no CR in which patients received general medical care (7/19) and usual care (6/19), both of which are variable across settings, limiting external validity.^21 22 24 25 29 30 33–38 40^

### Methods

The five modelling studies used multiple evidence sources with likely mixed population characteristics.^34–38^ Only one study clearly described the identification of inputs.^37^ Four studies were Markov models which reflected the main outcomes identified in the clinical literature (active disease with varying symptoms, hospitalisation and death).^34 35 37 38^ One study did not clearly report methods.^36^ A single study presented a within-trial evaluation with an added survival model to extrapolate evidence over longer time horizons.^21^

Thirteen studies were trial-based economic evaluations. While trials are generally a robust evidence source, populations may act differently out of a trial, limiting generalisability. Eleven were randomised controlled trials (RCTs).^21 23–27 29–33 40^ One study was non-randomised; randomisation was proposed to participants but they could reject it and be allocated to the group of their choice.^28^ Randomisation was rejected by the majority, leaving the study susceptible to self-selection bias. Blinding was not explicitly addressed in most studies.^23 24 26–30 33 40^ One study reported that it was not blinded.^31^ The remaining studies mentioned that only researchers and practitioners were blinded.^21 25 32^ While blinding is an important factor in limiting bias, it is accepted that blinding is challenging in non-pharmacological trials.^41^

One study used a large Medicare database for a retrospective cohort study.^22^ Although more susceptible to bias than RCT evidence, this approach has some advantages, for example, cohort size (n=4324).^22^

### Health benefit

The majority of cost–utility studies used quality-adjusted life-years (QALYs) that incorporated data from generic survey based measures of health status: the EuroQol-5D  (EQ-5D) (7/16),^24 25 27 28 32 34 37^ 36-Item Short Form Health Survey (2/16)^26 31^ and the Assessment of Quality of Life 4D questionnaire (1/16).^38^ The EQ-5D has been shown to be a valid/reliable measure in the cardiovascular population and is recommended in English guidelines.^42 43^ One study used a disease-specific questionnaire, that is, the Utility-based Quality of Life-Heart Questionnaire.^30^ Two studies used the time trade-off method to estimate utility.^33 35^ The time trade-off is a direct utility measurement, whereas survey measures are indirect. While both methods (direct and indirect) are robust, the literature has noted that values produced differ between the methods, limiting comparability between studies.^44^ One study found that the use of direct measurement increased the net QALY and cost-effectiveness of the intervention compared with a generic measure, highlighting that caution is needed when interpreting results across studies due to the variation in methods.^23^

All the cost-effectiveness studies used life-years as the outcome.^21 22 35 37^ This ignores one of the key goals of CR (reducing morbidity), potentially underestimating the benefits of an effective intervention.

### Costs

Two studies obtained costs from cost databases without specifying the types of costs included.^22 34^ Costs included in the remaining studies are shown in table 2. Three studies only included cardiac-related costs, neglecting possible interactions between cardiac health and general health.^32 35 38^ Lost wages/productivity losses are less relevant in this population; a UK CR audit reported the mean age for accessing CR to be above retirement age.^4^ More relevant are informal care costs (1/19) as family/friends may undertake caring responsibilities. However, informal care data may be difficult to collect and highly variable.

**Table 2:** Included costs

Type of cost	Study references	Proportion of studies (n)
Healthcare costs		
Intervention	21 23–33 35–38 40	89% (17/19)
Hospitalisation	21 23 24 26–33 35–38 40	84% (16/19)
Outpatient	23 24 26–33 35–38 40	79% (15/19)
Primary/community care	23 24 26 27 29 31 33 38	42% (8/19)
Medication	27 30 31 37	21% (4/19)
Other costs		
Patient out of pocket	23 24 27 29 30	26% (5/19)
Lost wages to attend CR sessions	21 24 29 36	21% (4/19)
Productivity losses associated with illness	28 29 36	16% (3/19)
Informal care	36	5% (1/19)

CR,  cardiac rehabilitation.

### Uncertainty analysis

The majority of studies (17/19) included some sensitivity analysis.^21–30 32–35 37 38 40^ Fifteen studies included a probabilistic sensitivity analysis (PSA) using Monte Carlo simulation to judge uncertainty^22–29 32–35 37 38 40^ In nine of the papers, authors explicitly compared PSA results with a threshold for cost-effectiveness.^23–25 29 33 35 37 38 40^ In a further six studies, PSA was conducted, but authors did not define a threshold.^22 26–28 32 34^ Nine studies included PSA and one-way sensitivity analysis,^26–29 33–35 37 38^ whereas  two studies only included one-way sensitivity analysis.^21 30^ The most common parameters varied in one-way sensitivity analysis were intervention and hospitalisation costs, mortality rates and repeat events, and utilities. A single study considered subgroups, analysing results separately according to cardiac risk, referring diagnosis and gender.^33^

Completed Drummond checklists are given in the online supplementary material.

### Study results

Table 3 displays the key study results.

**Table 3:** Study results

Study	Intervention and comparator	Net health benefits (per patient)	Net costs (per patient)	Incremental cost-effectiveness ratio	Probability of cost-effectiveness
			Updated to common currency		
Comparing CR with no CR					
Georgiou *et al*, 2001^21^	Long-term moderate exercise training versus no exercise training	1.82 LYG	$4650	$2555/life-year saved	NR
Briffa *et al*, 2005^30^	Comprehensive CR plus UC versus no CR	0.009 QALYs	$392	$42 233/QALY	NR
Huang *et al*, 2008^22^	CR versus no CR	76 days life expectancy	$4276	$20 447/life-year saved	NR
Oldridge *et al*, 2008^23^	CR versus no CR	0.011 QWB-derived QALYs	$789	$71 755 per QALY (QWB derived QALYs)	58% (QWB-derived QALYs)
		0.040 TTO-derived QALYs		$19 740 per QALY (patient TTO-derived QALYs)	83% (TTO-derived QALYs)
Leggett *et al*, 2015^34^	Centre-based outpatient CR programme versus no CR	0.07 QALYs	$2147	$30 943/QALY	NR
Rincón e*t al*, 2016^35^	Exercise-based CR plus UC versus no CR programme	0.009 LYG	$312	$3367/LYG	76%
		0.29 QALYs		$1065/QALY	
De Gruyter *et al*, 2016^36^	50% CR uptake (scenario 1) versus 30% uptake	NR	NR	BCR of 5.6	NR
	65% CR uptake (scenario 2) versus 30% uptake	NR	NR	BCR of 6.8	NR
Comparing exercise components of CR with education					
Yu *et al*, 2004^31^	CR and prevention programme (exercise and education) versus usual care (education only)	0.6 QALYs	−$527	Dominant	NR
Reed *et al*, 2010^24^	Exercise training plus UC versus UC (education only)	0.03 QALYs	−$2938 (adjusted for baseline characteristics)	Varied between dominant and $43 141/QALY	59%–74%
			$1294 (including patient time and out-of-pocket costs)		
Kühr *et al*, 2011^37^	Supervised exercise therapy alongside standard care versus standard care	0.13 LYG	$2911	$23 598/LYG	55%
		0.10 QALYs		$29 498/QALY	
Comparing telehealth interventions with CR based in a healthcare centre					
Cheng *et al*, 2016^38^	Healthy weight intervention (pedometer based) versus UC	0.04 QALYs (men)	$1092 (men)	$3287/QALY (men)	53%
		0.04 QALYs (women)	$973 (women)	$2630/QALY (women)	
	Physical activity intervention (pedometer based) versus UC	0.80 QALYs (men)	$1789 (men)	$2227/QALY (men)	46%
		0.88 QALYs (women)	$1625 (women)	$1854/QALY (women)	
Maddison *et al*, 2015^25^	Heart exercise and remote technologies mobile phone intervention plus UC versus UC (exercise and cardiac support group)	NR	$203†	$24 385/QALY	72%–90%
Frederix *et al*, 2016^32^	Cardiac telerehabilitation programme in addition to conventional centre-based CR versus centre-based CR programme	0.026 QALYs	−$616	Dominant	NR
Kidholm *et al*, 2016^26^	ICT delivered individualised cardiac telerehabilitation programme versus traditional rehabilitation programme at the hospital or healthcare centre	0.004 QALYs	$2029	$588 734/QALY	NR
Comparing distribution of CR programmes					
Papadakis *et al*, 2008^33^	CR programme distributed over 12 months versus standard CR over 3 months	0.009 QALYs	−$131	Dominant	63%–67%
Comparing care settings of CR programmes					
Taylor *et al*, 2007^27^	Home-based CR versus hospital-based rehabilitation	−0.06 QALYs	$186	−$3092/QALY	NR
Schweikert *et al*, 2009^28^	Outpatient CR versus inpatient CR	0.048 QALYs	−$4200	Dominant	NR
Comparing psychological intervention with usual care					
Lewin *et al*, 2009^40^	Home-based cognitive–behavioural programme versus UC	NR	−$32	Dominant	67%
Dehbarez *et al*, 2015^29^	Learning and coping education strategies versus US (standard CR)	0.005 QALYs	$1131	$226 128/QALY	29%

Net costs and net health benefits reflect the time horizon adopted by the study, thus these should only be used to demonstrate whether interventions were cost saving or increasing, and whether they improved health or not.

BCR, Benefit Cost Ratio; CR, cardiac rehabilitation; ICT, information and communication technology; LYG, life-year gained; NR, not reported; TTO, Time Trade Off; QALY, quality-adjusted life-year;QWB, Quality of Well-being, UC, usual care.

#### General CR

All studies had a positive net cost (higher costs in the intervention arm) and were associated with an increase in health, hence it is up to decision-makers to decide whether the cost increase is worth the health gains. All studies comparing CR with no CR judged that the intervention was cost-effective at chosen thresholds (thresholds differ across countries and papers reflected this).^45^ A threshold does not exist for life-year gained (LYG) which limits the interpretation. Interpreting the cost–benefit study results is challenging due to the reporting in the paper (eg, no specified monetary value attributed to a disability adjusted life-year) and the lack of clear consensus on how to interpret cost–benefit analysis.

Two studies that considered exercise-based CR versus no CR produced the lowest ICER values per QALY and LYGs.^21 35^ Although this suggests that exercise-focused CR may be the most cost-effective option, differences in study design means that this conclusion is limited and uncertain.

One study focused on CR uptake rates and concluded that higher uptake rates would reduce disease burden; however, the use of RCT data to inform outcomes may not reflect real life.^36^

Two of the studies looking at CR versus no CR included PSA assessing the probability of cost-effectiveness given uncertainty in the data used. Estimates ranged between 58% and 83%.^23 35^

#### Exercise components of CR

Two studies compared exercise-based CR with an education only option.^24 31^ Depending on the costing perspective, both studies had an instance where intervention dominated education only (cost saving and health increasing). Both studies concluded exercise-based CR was cost-effective. One of the studies included uncertainty analysis, estimating that exercise-based CR was cost-effective in 59%–74% of cases.^24^ A further study compared supervised exercise therapy with standard care and concluded that it would be cost-effective in around 55% of cases, demonstrating uncertainty.^37^.

#### Telehealth-based or assisted CR

Four of the most recent studies looked at the use of telehealth interventions.^25 26 32 38^ One study comparing an individualised ICT delivered CR with hospital-based CR produced the highest ICER estimate identified across all of the studies ($588 734 per QALY).^26^ However, authors noted that in real life, increasing patient numbers may increase economies of scale and reduce costs. The remaining studies compared telehealth rehabilitation packages that tracked health and exercise statistics.^25 32 38^ All studies concluded that telehealth interventions considered were cost-effective (from dominant to $24 385 per QALY). Estimates of the probability of cost-effective were very different; in one study this ranged between 46% and 53% indicating substantial uncertainty, whereas in the other study it was between 72% and 90%.^25 38^

#### Other studies

Distribution of CR over longer or shorter time frames was the focus of one study, which found that a 12-month programme was dominant and cost-effective in over 60% of cases versus 3 months.^33^ This was the only study to look at results in different subgroups, finding that the 12-month CR was cost-effective for patients with lower risks of disease progression and for female patients, whereas 3-month CR was cost-effective for high-risk patients and for male patients. Two studies compared CR settings; no significant differences were found between home-based and hospital-based CR, but outpatient CR was found to be cost-effective than inpatient CR.^27 28^ The two studies on psychological therapy had very different findings, likely due to the wide variation in intervention type: with one study identifying a home-based cognitive–behavioural programme to be cost-effective in the majority of cases versus usual care (67%) and the other finding that learning and coping education strategies to be cost-effective in only 29% of the time.^29 40^

## Discussion

This review evaluates the cost-effectiveness of CR in the modern era providing a fresh evidence base for policy-makers. The majority of studies concluded that CR was cost-effective versus no CR, but there was more variation in the results of studies focusing on single components or delivery of CR. Exercise intervention in CR appears cost-effective, though uncertainty was high. Evidence for psychological intervention was limited and varied. Telehealth was the focus of recently published papers and the evidence found is in alignment with the wider literature; although there is evidence of cost-effectiveness, larger, robust studies are needed to strengthen conclusions^46^ 47 48. There is also the likelihood that telehealth results in a real-world setting may be very different; for example, economics of scale may reduce costs, patients may adhere to the technologies differently and for uncertain lengths of time.

Key drivers of cost-effectiveness were risk of subsequent events and hospitalisation, hospitalisation and intervention costs, and utilities. Only one study considered results by subgroups: identifying differences according to gender, referral reason and cardiac risk.^33^ Given the variation in patients referred to CR, this suggests not all patients should be treated in the same way. All studies had limitations including uncertainty, sample sizes and data sources. Combined with heterogeneity across methods, population and settings, the evidence is uncertain.

Our review took a different approach in terms of study inclusion to previous reviews, focusing on full economic evaluations across all CR groups, intervention types and settings. Although previous reviews found evidence to support the cost-effectiveness of CR intervention, this included the use of data from the 1980s to 1990s which struggles to speak to the challenges of healthcare commissioning in the modern era of cardiology.^6 7^ Similar to our review, authors noted that evidence was limited by study quality, variation in CR design/delivery and uncertainty.^6 7^

This review is subject to limitations. It was limited to English-language articles, introducing a risk of bias. Searches did not include the grey literature, therefore may be less likely to identify studies with uncertain or negative findings.^49^ The evidence base should be re-evaluated over time as new papers are published.

This review highlights specific areas for subsequent studies to investigate and address, particularly uncertainty due to study design and data, definitions for standard care and subgroup analyses. The review also indicates a paucity of evidence in low/middle-income countries, despite 80% of cardiovascular-related deaths occurring in these countries.^50^ Finally, despite evidence linking symptoms of anxiety and depression to repeat events and mortality, psychological interventions were the least common component of CR considered and results were mixed. Future research is needed to determine whether and what forms of psychological therapy could be cost-effective in CR. This should be prioritised given recent calls for closer integration of psychological and physical health outcomes.^51^

## References

[1] GBD 2015 Disease and Injury Incidence and Prevalence Collaborators. Global, regional, and national incidence, prevalence, and years lived with disability for 310 diseases and injuries, 1990-2015: a systematic analysis for the Global Burden of Disease Study 2015. Lancet 2016;388:1545–602. 10.1016/S0140-6736(16)31678-6 27733282 PMC5055577

[2] British Association for Cardiovascular Prevention and Rehabilitation. The six core components for cardiovascular disease prevention and rehabilitation. 2017 http://www.bacpr.com/resources/AC6_BACPRStandards&CoreComponents2017.pdf (accessed 26 Nov 2017).

[3] Dalal HM, Doherty P, Taylor RS Cardiac rehabilitation. BMJ 2015;351:h5000 10.1136/bmj.h5000 26419744 PMC4586722

[4] British Heart Foundation. The national audit of cardiac rehabilitation annual statistical report. 2016 http://www.cardiacrehabilitation.org.uk/docs/BHF_NACR_Report_2016.pdf (accessed 7 Apr 2017).

[5] NHS England. A resource to support commissioners in setting a level of ambition on reducing premature mortality prepared by medical directorate, NHS england factsheet: increase uptake of cardiac rehabilitation for people with coronary artery disease and following acut. 2014 https://www.england.nhs.uk/wp-content/uploads/2014/02/pm-fs-3-10.pdf (accessed 22 Jul 2017).

[6] Wong WP, Feng J, Pwee KH, et al A systematic review of economic evaluations of cardiac rehabilitation. BMC Health Serv Res 2012;12:243 10.1186/1472-6963-12-243 22873828 PMC3465180

[7] Papadakis S, Oldridge NB, Coyle D, et al Economic evaluation of cardiac rehabilitation: a systematic review. Eur J Cardiovasc Prev Rehabil 2005;12:513–20. 16319539 10.1097/01.hjr.0000186624.60486.e8

[8] Oldridge NB, Pakosh MT, Thomas RJ Cardiac rehabilitation in low- and middle-income countries: a review on cost and cost-effectiveness. Int Health 2016;8:77–82. 10.1093/inthealth/ihv047 26208507

[9] Rauch B, Davos CH, Doherty P, et al The prognostic effect of cardiac rehabilitation in the era of acute revascularisation and statin therapy: a systematic review and meta-analysis of randomized and non-randomized studies - The Cardiac Rehabilitation Outcome Study (CROS). Eur J Prev Cardiol 2016;23:1914–39. 10.1177/2047487316671181 27777324 PMC5119625

[10] Anderson L, Taylor RS Cardiac rehabilitation for people with heart disease: an overview of Cochrane systematic reviews Cochrane database of systematic reviews. Chichester, UK: John Wiley & Sons, Ltd, 2014. 10.1002/14651858.CD011273.pub2 PMC7087435 25503364

[11] Centre for reviews and dissemination. 2014 http://www.crd.york.ac.uk/crdweb/searchstrategies.asp (accessed 21 Jul 2016).

[12] Scottish Intercollegiate Guidelines Network. Scottish intercollegiate guidelines network cardiac rehabilitation. https://www.scotphn.net/wp-content/uploads/2015/11/Cardiac_Rehabilitation.pdf (accessed 5 Nov 2017).

[13] Taylor RS, Dalal H, Jolly K, et al Home-based versus centre-based cardiac rehabilitation Cochrane database of systematic reviews. Chichester, UK: John Wiley & Sons, Ltd, 2015. 10.1002/14651858.CD007130.pub3 26282071

[14] National Institute for Health and Care Excellence. Chronic heart failure in adults: management | Guidance and guidelines | NICE. 2010 https://www.nice.org.uk/guidance/cg108/resources/cardiac-rehabilitation-services-commissioning-guide-304110253/chapter/3-assessing-service-levels-for-cardiac-rehabilitation-services (accessed 9 Mar 2017). 30645061

[15] National Institute for Health and Care Excellence. Myocardial infarction: cardiac rehabilitation and prevention of further cardiovascular disease. 2013 https://www.nice.org.uk/guidance/cg172 (accessed 26 Nov 2017). 31891465

[16] National Institute for Health and Care Excellence. Unstable angina and NSTEMI: early management. 2013 https://www.nice.org.uk/guidance/cg94 (accessed 26 Nov 2017). 32065742

[17] Drummond MF, Jefferson TO Guidelines for authors and peer reviewers of economic submissions to the BMJ. The BMJ economic evaluation working party. BMJ 1996;313:275. 8704542 10.1136/bmj.313.7052.275 PMC2351717

[18] Centre for Reviews and Dissemination. NHS Economic Evaluation Database (NHS EED) handbook. 2007 http://www.york.ac.uk/inst//crd/pdf/nhseed-handbook2007.pdf (accessed 18 Dec 2015).

[19] OECD. Prices - Inflation (CPI) - OECD data. 2015 https://data.oecd.org/price/inflation-cpi.htm (accessed 18 Dec 2015).

[20] OECD. Purchasing Power Parities (PPPs) data. 2015 http://www.oecd.org/std/prices-ppp/purchasingpowerparitiespppsdata.htm (accessed 18 Dec 2015).

[21] Georgiou D, Chen Y, Appadoo S, et al Cost-effectiveness analysis of long-term moderate exercise training in chronic heart failure. Am J Cardiol 2001;87:984–8. 10.1016/S0002-9149(01)01434-5 11305991

[22] Huang Y, Zhang R, Culler SD, et al Costs and effectiveness of cardiac rehabilitation for dialysis patients following coronary bypass. Kidney Int 2008;74:1079–84. 10.1038/ki.2008.381 18650790 PMC2777679

[23] Oldridge N, Furlong W, Perkins A, et al Community or patient preferences for cost-effectiveness of cardiac rehabilitation: does it matter? Eur J Cardiovasc Prev Rehabil 2008;15:608–15. 10.1097/HJR.0b013e328304fec1 18800005

[24] Reed SD, Whellan DJ, Li Y, et al Economic evaluation of the HF-ACTION (Heart Failure: A Controlled Trial Investigating Outcomes of Exercise Training) randomized controlled trial: an exercise training study of patients with chronic heart failure. Circ Cardiovasc Qual Outcomes 2010;3:374–81. 10.1161/CIRCOUTCOMES.109.907287 20551371 PMC3050585

[25] Maddison R, Pfaeffli L, Whittaker R, et al A mobile phone intervention increases physical activity in people with cardiovascular disease: Results from the HEART randomized controlled trial. Eur J Prev Cardiol 2015;22:701–9. 10.1177/2047487314535076 24817694

[26] Kidholm K, Rasmussen MK, Andreasen JJ, et al Cost-utility analysis of a cardiac telerehabilitation program: the teledialog project. Telemed J E Health 2016;22:553–63. 10.1089/tmj.2015.0194 26713491 PMC4939376

[27] Taylor RS, Watt A, Dalal HM, et al Home-based cardiac rehabilitation versus hospital-based rehabilitation: a cost effectiveness analysis. Int J Cardiol 2007;119:196–201. 10.1016/j.ijcard.2006.07.218 17084927

[28] Schweikert B, Hahmann H, Steinacker JM, et al Intervention study shows outpatient cardiac rehabilitation to be economically at least as attractive as inpatient rehabilitation. Clin Res Cardiol 2009;98:787–95. 10.1007/s00392-009-0081-6 19821135

[29] Dehbarez NT, Lynggaard V, May O, et al Learning and coping strategies versus standard education in cardiac rehabilitation: a cost-utility analysis alongside a randomised controlled trial. BMC Health Serv Res 2015;15:422 10.1186/s12913-015-1072-0 26412226 PMC4586001

[30] Briffa TG, Eckermann SD, Griffiths AD, et al Cost-effectiveness of rehabilitation after an acute coronary event: a randomised controlled trial. Med J Aust 2005;183:450. 16274344 10.5694/j.1326-5377.2005.tb07121.x

[31] Yu CM, Lau CP, Chau J, et al A short course of cardiac rehabilitation program is highly cost effective in improving long-term quality of life in patients with recent myocardial infarction or percutaneous coronary intervention. Arch Phys Med Rehabil 2004;85:1915–22. 15605326 10.1016/j.apmr.2004.05.010

[32] Frederix I, Hansen D, Coninx K, et al Effect of comprehensive cardiac telerehabilitation on one-year cardiovascular rehospitalization rate, medical costs and quality of life: A cost-effectiveness analysis. Eur J Prev Cardiol 2016;23:674–82. 10.1177/2047487315602257 26289723

[33] Papadakis S, Reid RD, Coyle D, et al Cost-effectiveness of cardiac rehabilitation program delivery models in patients at varying cardiac risk, reason for referral, and sex. Eur J Cardiovasc Prev Rehabil 2008;15:347–53. 10.1097/HJR.0b013e3282f5ffab 18525392

[34] Leggett LE, Hauer T, Martin B-J, et al Optimizing value from cardiac rehabilitation. Mayo Clin Proc 2015;90:1011–20. 10.1016/j.mayocp.2015.05.015 26149321

[35] Rincón M, Rojas MX, Rodriguez Romero VA, et al Economic evaluation of exercise-based cardiac rehabilitation programs for chronic heart failure patients in Colombia. J Cardiopulm Rehabil Prev 2016;36:12–19. 10.1097/HCR.0000000000000150 26702862

[36] De Gruyter E, Ford G, Stavreski B Economic and social impact of increasing uptake of cardiac rehabilitation servicesa cost benefit analysis. Heart Lung Circ 2016;25:175–83. 10.1016/j.hlc.2015.08.007 26442971

[37] Kühr EM, Ribeiro RA, Rohde LEP, et al Cost-effectiveness of supervised exercise therapy in heart failure patients. Value in Health 2011;14:S100–S107. 10.1016/j.jval.2011.05.006 21839879

[38] Cheng Q, Church J, Haas M, et al Cost-effectiveness of a population-based lifestyle intervention to promote healthy weight and physical activity in non-attenders of cardiac rehabilitation. Heart Lung Circ 2016;25:265–74. 10.1016/j.hlc.2015.07.002 26669813

[39] Reed C, Monz BU, Perahia DG, et al Quality of life outcomes among patients with depression after 6 months of starting treatment: results from FINDER. J Affect Disord 2009;113:296–302. 10.1016/j.jad.2008.05.021 18603303

[40] Lewin RJ, Coulton S, Frizelle DJ, et al A brief cognitive behavioural preimplantation and rehabilitation programme for patients receiving an implantable cardioverter-defibrillator improves physical health and reduces psychological morbidity and unplanned readmissions. Heart 2009;95:63–9. 10.1136/hrt.2007.129890 18070951

[41] Boutron I, Tubach F, Giraudeau B, et al Blinding was judged more difficult to achieve and maintain in nonpharmacologic than pharmacologic trials. J Clin Epidemiol 2004;57:543–50. 10.1016/j.jclinepi.2003.12.010 15246122

[42] Dyer MT, Goldsmith KA, Sharples LS, et al A review of health utilities using the EQ-5D in studies of cardiovascular disease. Health Qual Life Outcomes 2010;8:13 10.1186/1477-7525-8-13 20109189 PMC2824714

[43] National Institute for Health and Care Excellence. Guide to the methods of technology appraisal 2013. 2013 https://www.nice.org.uk/process/pmg9/chapter/foreword (accessed 10 Feb 2016). 27905712

[44] Arnold D, Girling A, Stevens A, et al Comparison of direct and indirect methods of estimating health state utilities for resource allocation: review and empirical analysis. BMJ 2009;339:b2688 10.1136/bmj.b2688 22128393 PMC2714630

[45] Shiroiwa T, Sung YK, Fukuda T, et al International survey on willingness-to-pay (WTP) for one additional QALY gained: what is the threshold of cost effectiveness? Health Econ 2010;19:422–37. 10.1002/hec.1481 19382128

[46] Grustam AS, Severens JL, van Nijnatten J, et al Cost-effectiveness of telehealth interventions for chronic heart failure patients: a literature review. Int J Technol Assess Health Care 2014;30:59–68. 10.1017/S0266462313000779 24495581

[47] Udsen FW, Hejlesen O, Ehlers LH A systematic review of the cost and cost-effectiveness of telehealth for patients suffering from chronic obstructive pulmonary disease. J Telemed Telecare 2014;20:212–20. 10.1177/1357633X14533896 24803277

[48] de la Torre-Díez I, López-Coronado M, Vaca C, et al Cost-utility and cost-effectiveness studies of telemedicine, electronic, and mobile health systems in the literature: a systematic review. Telemed J E Health 2015;21:81–5. 10.1089/tmj.2014.0053 25474190 PMC4312789

[49] Bell CM, Urbach DR, Ray JG, et al Bias in published cost effectiveness studies: systematic review. BMJ 2006;332:699–703. 10.1136/bmj.38737.607558.80 16495332 PMC1410902

[50] Fuster V Global burden of cardiovascular disease. J Am Coll Cardiol 2014;64:520–2. 10.1016/j.jacc.2014.06.1151 25082587

[51] Das P, Naylor C, Majeed A, et al Bringing together physical and mental health within primary care: a new frontier for integrated care. J R Soc Med 2016;109:364–6. 10.1177/0141076816665270 27729592 PMC5066536

