# Supplementary material for: Cost-effectiveness of cardiac rehabilitation: a systematic review
Source: Heart. 2018 Apr 13;104(17):1403–10. doi: 10.1136/heartjnl-2017-312809 (PMC6109236; doi:10.1136/heartjnl-2017-312809)
Supplement: Supplementary file 1 [file heartjnl-2017-312809supp001.pdf]

Supplementary material

Search strategies

The Cochrane Library strategy

Please note, no economic search terms are required in this strategy because narrowing to the NHS EED and HTA databases will capture relevant study types.

1. cardiac rehabilitation:ti,ab Publication Year from 2000 to 2017
2. MeSH descriptor: [Myocardial Infarction] explode all trees
3. MeSH descriptor: [Angina Pectoris] explode all trees
4. congestive heart failure:ti,ab
5. congenital heart defect:ti,ab
6. heart valve diseases:ti,ab
7. rheumatic heart disease:ti,ab
8. MeSH descriptor: [Heart Transplantation] explode all trees
9. angioplasty, transluminal, percutaneous coronary:ti,ab
10. coronary disease:ti,ab
11. cardiovascular diseases:ti,ab
12. heart diseases:ti,ab
13. coronary artery bypass:ti,ab
14. heart disease*:ti,ab
15. myocard* infarc*:ti,ab
16. coronary artery disease:ti,ab
17. acute coronary syndrome:ti,ab
18. percutaneous coronary intervention:ti,ab
19. unstable angina:ti,ab
20. chronic heart failure:ti,ab
21. implantable cardiac defibrillat*:ti,ab
22. #2 or #3 or #4 or #5 or #6 or #7 or #8 or #9 or #10 or #11 or #12 or #13 or #14 or #15 or #16 or #17 or #18 or #19 or #20 or #21
23. MeSH descriptor: [Rehabilitation] explode all trees
24. rehab*:ti,ab
25. rehabilitation:ti,ab
26. #23 or #24 or #25
27. #22 and #26 Publication Year from 2005 to 2015, in Technology Assessments and Economic Evaluations
28. #1 or #27

MEDLINE strategy

1. (cardiac adj rehab$).mp
2. exp cardiovascular diseases/rh
3. exp myocardial infarction/
4. mi.tw
5. myocardial ischemia/
6. exp angina pectoris/
7. exp heart failure, congestive/
8. exp heart defects, congenital/
9. exp heart valve diseases/
10. rheumatic heart disease/
11. exp heart transplantation/
12. angioplasty, transluminal, percutaneous coronary/
13. ptca.tw
14. coronary disease/
15. cardiovascular diseases/
16. heart diseases/

Supplementary material

17. coronary artery bypass/
18. cabg.tw
19. (heart adj disease$).mp
20. (myocard$ adj infarct$).mp
21. coronary artery disease/
22. acute coronary syndrome/
23. percutaneous coronary intervention/
24. PCI.tw
25. stent.tw
26. unstable angina/
27. chronic heart failure/
28. CHF.tw
29. (implantable cardiac defibrillat$).mp
30. ICD.tw
31. or/3-30
32. rehabilitation/
33. (rehabilitation cent&).mp
34. rehabilitation nursing/
35. rehab$.tw
36. or/32-35
37. 1 or 2 or (31 and 36)
38. Economics/
39. exp "costs and cost analysis"/
40. Economics, Dental/
41. exp economics, hospital/
42. Economics, Medical/
43. Economics, Nursing/
44. Economics, Pharmaceutical/
45. (economic$ or cost or costs or costly or costing or price or prices or pricing or pharmacoeconomic$).ti,ab.
46. (expenditure$ not energy).ti,ab.
47. value for money.ti,ab.
48. budget$.ti,ab.
49. or/38-48
50. ((energy or oxygen) adj cost).ti,ab.
51. (metabolic adj cost).ti,ab.
52. ((energy or oxygen) adj expenditure).ti,ab.
53. or/50-52
54. 49 not 53
55. letter.pt.
56. editorial.pt.
57. historical article.pt.
58. or/55-57
59. 54 not 58
60. exp animals/ not humans/
61. 59 not 60
62. limit 61 to yr="2014 -Current"
63. 37 and 62

PsycINFO strategy

Supplementary material

1. (cardiac adj2 rehab$).ti,ab.
2. (cardiovascular adj2 diseas$).ti,ab.
3. (myocardial infarction).ti,ab.
4. mi.mp.
5. (myocardial ischemia).ti,ab.
6. (angina pectoris).ti,ab.
7. (congestive heart failure).ti,ab.
8. (congenital heart defects$).ti,ab.
9. (heart valve diseases).ti,ab.
10. (rheumatic heart disease).ti,ab.
11. (heart transplantation).ti,ab.
12. (angioplasty, transluminal, percutaneous coronary).ti,ab.
13. ptca.mp.
14. (coronary disease).ti,ab.
15. (cardiovascular diseases).ti,ab.
16. (heart diseases).ti,ab.
17. (coronary artery bypass).ti,ab.
18. cabg.mp.
19. (heart adj2 disease$).ti,ab.
20. (myocard$ adj2 infarct$).ti,ab.
21. (coronary artery disease).ti,ab.
22. (acute coronary syndrome).ti,ab.
23. (percutaneous coronary intervention).ti,ab.
24. PCI.mp.
25. Stent.mp.
26. (unstable angina).ti,ab.
27. (chronic heart failure).ti,ab.
28. CHF.mp.
29. (implantable cardiac defibrillat$).ti,ab.
30. ICD.mp.
31. or/3-30
32. rehabilitation.mp.
33. (rehabilitation cent&).ti,ab.
34. (rehabilitation nursing).ti,ab.
35. rehab$.mp.
36. or/32-35
37. 1 or 2 or (31 and 36)
38. "costs and cost analysis"/
39. "Cost Containment"/
40. (economic adj2 evaluation$).ti,ab.
41. (economic adj2 analy$).ti,ab.
42. (economic adj2 (study or studies)).ti,ab.
43. (cost adj2 evaluation$).ti,ab.
44. (cost adj2 analy$).ti,ab.
45. (cost adj2 (study or studies)).ti,ab.
46. (cost adj2 effective$).ti,ab.
47. (cost adj2 benefit$).ti,ab.
48. (cost adj2 utili$).ti,ab.
49. (cost adj2 minimi$).ti,ab.
50. (cost adj2 consequence$).ti,ab.
51. (cost adj2 comparison$).ti,ab.
52. (cost adj2 identificat$).ti,ab.
53. (pharmacoeconomic$ or pharmaco-economic$).ti,ab.
54. or/38-53
55. (task adj2 cost$).ti,ab,id.
56. (switch$ adj2 cost$).ti,ab,id.
57. (metabolic adj cost).ti,ab,id.
58. ((energy or oxygen) adj cost).ti,ab,id.
59. ((energy or oxygen) adj expenditure).ti,ab,id.
60. or/55-59

Supplementary material

61. (animal or animals or rat or rats or mouse or mice or hamster or hamsters or dog or dogs or cat or cats or bovine or sheep or ovine or pig or pigs).ab,ti,id,de.
62. editorial.dt.
63. letter.dt.
64. dissertation abstract.pt.
65. or/61-64
66. 54 not (60 or 65)
67. limit 66 to yr="2014 -Current"
68. 37 and 67

Embase strategy

1. (cardiac adj rehab$).mp
2. exp cardiovascular diseases/rh
3. exp myocardial infarction/
4. mi.tw
5. myocardial ischemia/
6. exp angina pectoris/
7. exp heart failure, congestive/
8. exp heart defects, congenital/
9. exp heart valve diseases/
10. rheumatic heart disease/
11. exp heart transplantation/
12. angioplasty, transluminal, percutaneous coronary/
13. ptca.tw
14. coronary disease/
15. cardiovascular diseases/
16. heart diseases/
17. coronary artery bypass/
18. cabg.tw
19. (heart adj disease$).mp
20. (myocard$ adj infarct$).mp
21. coronary artery disease/
22. acute coronary syndrome/
23. percutaneous coronary intervention/
24. PCI.tw
25. stent.tw
26. unstable angina/
27. chronic heart failure/
28. CHF.tw
29. (implantable cardiac defibrillat$).mp
30. ICD.tw
31. or/3-30
32. rehabilitation/
33. (rehabilitation cent&).mp
34. rehabilitation nursing/
35. rehab$.tw
36. or/32-35
37. 1 or 2 or (31 and 36)
38. Health Economics/
39. exp Economic Evaluation/
40. exp Health Care Cost/
41. pharmacoeconomics/
42. 38 or 39 or 40 or 41
43. (econom$ or cost or costs or costly or costing or price or prices or pricing or pharmacoeconomic$).ti,ab.
44. (expenditure$ not energy).ti,ab.
45. (value adj2 money).ti,ab.
46. budget$.ti,ab.
47. 43 or 44 or 45 or 46
48. 42 or 47

Supplementary material

49. letter.pt.
50. editorial.pt.
51. note.pt.
52. 49 or 50 or 51
53. 48 not 52
54. (metabolic adj cost).ti,ab.
55. ((energy or oxygen) adj cost).ti,ab.
56. ((energy or oxygen) adj expenditure).ti,ab.
57. 54 or 55 or 56
58. 53 not 57
59. animal/
60. exp animal experiment/
61. nonhuman/
62. (rat or rats or mouse or mice or hamster or hamsters or animal or animals or dog or dogs or cat or cats or bovine or sheep).ti,ab,sh.
63. 59 or 60 or 61 or 62
64. exp human/
65. human experiment/
66. 64 or 65
67. 63 not (63 and 66)
68. 58 not 67
69. conference abstract.pt.
70. 68 not 69
71. limit 70 to yr="2001 -Current"
72. 37 and 71

Drummond checklist

The Drummond checklist was comepleted for all studies, results are summarised within the paper. The full tables can be viewed below.

Key:

✓/✗= Unclear or addressed in part

✓ = Yes or addressed

✗= No or not addressed

NR = not reported

Supplementary material

Drummond's check-list for assessing economic evaluations. (Drummond M et al. Methods for the economic evaluation of health care programmes. 2nd ed. Oxford. Oxford University Press. 1997)	Georgiou et al. 2001	Briffa et al. 2005	Huang et al.2008	Oldridge et al. 2008	Leggett et al. 2015	Rincón et al. 2016	De Gruyter et al. 2016	Yu et al. 2004	Reed et al. 2010
**1. Was a well-defined question posed in answerable form?**	✓/✗	✓	✓/✗	✓	✓	✓	✓/✗	✓/✗	✓/✗
1.1. Did the study examine both costs and effects of the service(s) or programme(s)?	✓	✓	✓	✓	✓	✓	✓	✓	✓
1.2. Did the study involve a comparison of alternatives?	✓	✓	✓	✓	✓	✓	✓	✓	✓
1.3. Was a viewpoint for the analysis stated and was the study placed in any particular decision-making context?	✗Perspective unclear	✓	✗Perspective unclear	✓	✓	✓	✗Perspective unclear	✗Perspective unclear	✗Perspective unclear
**2. Was a comprehensive description of the competing alternatives given (i.e. can you tell who did what to whom, where, and how often)?**	✓Intervention<br>✗ Control	✓	✗	✓	✗	✓	✗	✓	✓Intervention<br>✗ Control
2.1. Were there any important alternatives omitted?	**N/A** It would be unfeasible to cover all components and designs of CR under a trial design	**N/A** It would be unfeasible to cover all components and designs of CR under a trial design	**N/A** It would be unfeasible to cover all components and designs of CR under a trial design	**N/A** It would be unfeasible to cover all components and designs of CR under a trial design	**N/A** Is a modelling study it could have been expanded to include a wider range of CR options, however, this is likely to be unfeasible due to limited data and uncertainty in the data	**N/A** As a modelling study it could have been expanded to include a wider range of CR options, however, this is likely to be unfeasible due to limited data and uncertainty in the data	**N/A** As a modelling study it could have been expanded to include a wider range of CR options, however, this is likely to be unfeasible due to limited data and uncertainty in the data	**N/A** It would be unfeasible to cover all components and designs of CR under a trial design	**N/A** It would be unfeasible to cover all components and designs of CR under a trial design
2.2. Was (should) a do-nothing alternative be considered?	✓ This was essentially the comparator (no CR)	✓ This was essentially the comparator (no CR)	✓ This was essentially the comparator (no CR)	✓ This was essentially the comparator (no CR)	✓ This was essentially the comparator (no CR)	✓ This was essentially the comparator (no CR)	✗Study focused on update rates, a 0% scenario was not included and would have been interesting	✗Likely to be justified as some CR standard care	✗Likely to be justified as some CR standard care
**3. Was the effectiveness of the programme or services established?**	✓	✓	✓	✓	✓	✓	✓	✓	✓
3.1. Was this done through a randomised, controlled clinical trial? If so, did the trial protocol reflect what would happen in regular practice?	✓	✓	✗	✓ RCT data, however data from 1994 which reduces validity	✗	✗	✗Modelling study (parameters taken from the literature)	✓	✓
3.2. Was effectiveness established through an overview of clinical studies?	✗Single RCT	✓/✗In part, mortality was taken from a meta-analysis of published RCT	✗Single RCT	✗Single RCT	✗Single RCT	✓Authors obtained data from existing review (including a meta-analysis of	✓/✗Readmissions were taken from a review. Mortality was taken from a single study (an old	✗Single RCT	✗Single RCT

Supplementary material

		evidence. Remaining effectiveness inputs were taken from the RCT				RCT evidence) and described how this was identified	source, 1988, which impacts validity). In addition, authors did not report how the literature was identified		
3.3. Were observational data or assumptions used to establish effectiveness? If so, what are the potential biases in results?	✗	✗	✓ Restrospective data from claims databases, therefore it is susceptible to bias as not randomized or blinded. Groups were not similar at baseline which also introduces further potential bias	✗	✓Retrospectively gathered data from a database of outcomes in CHD	✗	✗	✗	✗
**4. Were all the important and relevant costs and consequences for each alternative identified?**	✓/✗	✓/✗	✓/✗	✓	✓/✗	✓/✗	✓/✗	✓	✓
4.1. Was the range wide enough for the research question at hand?	✓ Costs<br>✗ Outcomes as no measure of HRQoL or utility included)	✓	✗ Outcomes as no measure of HRQoL or utility included<br>✗ Costs - authors used a claims database and costs specified	✓	✓ Outcomes<br>✗ Costs - authors used a claims database and costs specified	✓ Costs<br>✗ Outcomes as no measure of HRQoL or utility included)	✓ Outcomes<br>✗Costs do not include primary care	✓/✗<br>Unclear as the perspective was not stated. However, covers key direct costs	✓
4.2. Did it cover all relevant viewpoints? (Possible viewpoints include the community or social viewpoint, and those of patients and third-party payers. Other viewpoints may also be relevant depending upon the particular analysis.)	✓ For the stated perspective, however whilst the authors include lost wages to attend, lost wages due to disability are not incorporated	✓ For the stated perspective, though the exclusion of primary healthcare costs may underestimate total costs	✓/✗Unsure due to the lack of reporting associated with the use of claims data	✓	✓/✗Unsure due to the lack of reporting associated with the use of a database	✓ For the stated perspective, though the exclusion non-cardiac related cost is a narrow perspective	✓/✗Unsure as perspective was not stated, likely not as primary care costs were not included	✓ With the exception of societal costs	✓
4.3. Were the capital costs, as well as operating costs, included?	✓	✓	**NR**	✓	**NR**	**NR**	**NR**	✓	✓
**5. Were costs and consequences measured accurately in appropriate physical units (e.g. hours of nursing time, number of physician visits, lost work-days, gained life years)?**	✓	✓	✓/✗<br>Unsure due to the lack of reporting associated with the use of claims data	✓	✓/✗<br>Unsure due to the lack of reporting associated with the use of claims data	✓	✓	✓	✓
5.1. Were any of the identified items omitted from measurement? If so, does this mean that they carried no weight in the subsequent analysis?	✗	✗	See above	✗	See above	✗	✗	✗	✗

Supplementary material

5.2. Were there any special circumstances (e.g., joint use of resources) that made measurement difficult? Were these circumstances handled appropriately?	**NR**	**NR**	See above	**NR**	See above	**NR**	**NR**	✗	**NR**
**6. Were the cost and consequences valued credibly?**	✓	✓	✓/✗	✓	✓/✗	✓/✗	✓	✓	✓
6.1. Were the sources of all values clearly identified? (Possible sources include market values, patient or client preferences and views, policy-makers' views and health professionals' judgements)	✓	✓ Although a meta-analysis was used to inform mortality and the authors do not state how they identified this	✓	✓	✓ However, it would have been more robust to explain how published estimates were applied in the model	✗Authors provide a reference, they do not clearly identify which parameters were taken from this and what the value of each was	✓Although it would be improved by specifying how the used literature was identified	✓	✓
6.2. Were market values employed for changes involving resources gained or depleted?	✓	**NR**	**NR**	✓	✓	**NR**	**NR**	✓	✓
6.3. Where market values were absent (e.g. volunteer labour), or market values did not reflect actual values (such as clinic space donated at a reduced rate), were adjustments made to approximate market values?	**NR**	**NR**	**NR**	✓	**NR**	**NR**	**NR**	**NR**	**NR**
6.4. Was the valuation of consequences appropriate for the question posed (i.e. has the appropriate type or types of analysis – cost-effectiveness, cost-benefit, cost-utility – been selected)?	✓	✓	✓	✓	✓	✓	✗Unclear as the methods are vague. It appears that it is a partial CBA as life years and DALYs have been included but not specifically related to costs	✓	✓
**7. Were costs and consequences adjusted for differential timing?**	✓	Not relevant due to time horizon	✓/✗	Not relevant due to time horizon	✓	**NR**	✓	**NR**	✓/✗
7.1. Were costs and consequences that occur in the future 'discounted' to their present values?	✓	See above	✓Costs discounted **NR** Outcomes	See above	✓	**NR**	✓	**NR**	✓Costs discounted **NR** Outcomes
7.2. Was there any justification given for the discount rate used?	✗	See above	✗	See above	✓	**NR**	✓	**NR**	✗
**8. Was an incremental analysis of costs and consequences of alternatives performed?**	✓ But the authors did not state an explicit threshold for cost-effectiveness	✓ Although they do not explicitly state the threshold for cost-effectiveness (instead noting that it is under the level accepted by the Australian system)	✓ In addition the authors state a threshold for cost-effectiveness	✓ Authors reported a threshold for cost-effectiveness	✓ Although authors did not state a threshold for cost-effectiveness	✓ The threshold for cost-effectiveness was also made explicit	✗	✓ However, the threshold was not stated	✓ The threshold for cost-effectiveness was also made explicit

Supplementary material

8.1. Were the additional (incremental) costs generated by one alternative over another compared to the additional effects, benefits, or utilities generated?	✓	✓	✓	✓	✓	✓	✗	✓	✓
**9. Was allowance made for uncertainty in the estimates of costs and consequences?**	✓ Although it was limited to one-way sensitivity analysis	✓ Although it was limited to one-way sensitivity analysis	✓ PSA, although only with a reported 1,000 simulations	✓ PSA	✓Comprehensive (one-way sensitivity analysis and PSA)	✓ Comprehensive (one-way sensitivity analysis and PSA)	✗	✗	✓PSA
9.1. If data on costs and consequences were stochastic (randomly determined sequence of observations), were appropriate statistical analyses performed?	✓	✓	✓	✓	✓	✓	**N/A**	**N/A**	✓
9.2. If a sensitivity analysis was employed, was justification provided for the range of values (or for key study parameters)?	✓	✗However, it appears that the authors tested a wide range and included key parameters	✗	✓	✓	✗	**N/A**	**N/A**	✓
9.3. Were the study results sensitive to changes in the values (within the assumed range for sensitivity analysis, or within the confidence interval around the ratio of costs to consequences)?	✓	✓	✓	✓	✓ Although confidence intervals were not reported	✓	**N/A**	**N/A**	✓
**10. Did the presentation and discussion of study results include all issues of concern to users?**	✓	✓/✗	✓/✗	✓/✗	✓/✗	✓/✗	✓/✗	✓/✗	✓/✗
10.1. Were the conclusions of the analysis based on some overall index or ratio of costs to consequences (e.g. cost-effectiveness ratio)? If so, was the index interpreted intelligently or in a mechanistic fashion?	✓	✓	✓	✓	✓	✓	✗	✗	✓
10.2. Were the results compared with those of others who have investigated the same question? If so, were allowances made for potential differences in study methodology?	✓	✓	✓	✓	✓	✓	✓	✓	✓
10.3. Did the study discuss the generalisability of the results to other settings and patient/client groups?	✓	✓	✗	✗	✓	✓	✓	✓	✓
10.4. Did the study allude to, or take account of, other important factors in the choice or decision under consideration (e.g. distribution of costs and consequences, or relevant ethical issues)?	✓	✓	✓	✗	✓	✓	✓	✓	✓

Supplementary material

10.5. Did the study discuss issues of implementation, such as the feasibility of adopting the 'preferred' programme given existing financial or other constraints, and whether any freed resources could be redeployed to other worthwhile programmes?	✓	✗	✗	✗	✗	✗	✗	✓	✗

**Drummond's check-list for assessing economic evaluations. (Drummond M et al. Methods for the economic evaluation of health care programmes. 2nd ed. Oxford. Oxford University Press. 1997)**	**Kühr et al. 2011**	**Cheng et al. 2016**	**Maddison et al. 2015**	**Frederix et al. 2016**	**Kidholm et al. 2016**	**Papadakis et al. 2008**	**Taylor et al. 2007**	**Schweikert et al. 2009**	**Lewin et al. 2009**	**Dehbarez et al. 2015**
**1. Was a well-defined question posed in answerable form?**	✓	✓	✓/✗	✓	✓	✓	✓/✗	✓	✓	✓
1.1. Did the study examine both costs and effects of the service(s) or programme(s)?	✓	✓	✓	✓	✓	✓	✓	✓	✓	✓
1.2. Did the study involve a comparison of alternatives?	✓	✓	✓	✓	✓	✓	✓	✓	✓	✓
1.3. Was a viewpoint for the analysis stated and was the study placed in any particular decision-making context?	✓	✓	✗Perspective unclear	✓	✓	✓	✗Perspective unclear	✓	✓	✓
**2. Was a comprehensive description of the competing alternatives given (i.e. can you tell who did what to whom, where, and how often)?**	✓Intervention ✗ Control	✓	✓	✓	✓	✓	✓	✓	✓	✓
2.1. Were there any important alternatives omitted?	**N/A** As a modelling study it could have been expanded to include a wider range of CR options, however, this is likely to be unfeasible due to limited data and uncertainty in the data	**N/A** As a modelling study it could have been expanded to include a wider range of CR options, however, this is likely to be unfeasible due to limited data and uncertainty in the data	**N/A** It would be unfeasible to cover all components and designs of CR under a trial design	**N/A** It would be unfeasible to cover all components and designs of CR under a trial design	**N/A** It would be unfeasible to cover all components and designs of CR under a trial design	**N/A** It would be unfeasible to cover all components and designs of CR under a trial design	**N/A** It would be unfeasible to cover all components and designs of CR under a trial design	**N/A** It would be unfeasible to cover all components and designs of CR under a trial design	**N/A** It would be unfeasible to cover all components and designs of CR under a trial design	**N/A** It would be unfeasible to cover all components and designs of CR under a trial design
2.2. Was (should) a do-nothing alternative be considered?	✗Likely to be justified as some CR standard care	✓ This was an option within the model	✗Likely to be justified as some CR standard care	✗Likely to be justified as some CR standard care	✗Likely to be justified as some CR standard care	✗Likely to be justified as some CR standard care	✗Likely to be justified as some CR standard care	✗Likely to be justified as some CR standard care	✗Likely to be justified as some CR standard care	✗Likely to be justified as some CR standard care
**3. Was the effectiveness of the programme or services established?**	✓	✓	✓	✓	✓	✓	✓	✓	✓	✓

Supplementary material

3.1. Was this done through a randomised, controlled clinical trial? If so, did the trial protocol reflect what would happen in regular practice?	✗This was a modelling study (parameters taken from the literature)	✓ RCT evidence used to inform the modelling approach and key data	✓ RCT evidence reflective of regular practise	✓ RCT evidence reflective of regular practise	✓ RCT evidence reflective of regular practise	✓ RCT evidence reflective of regular practise	✓ RCT evidence reflective of regular practise	✗Randomisation was proposed to participants; however they could decline and choose an arm. Only 2.5% of participants accepted randomisation	✓ RCT evidence	✓ RCT evidence
3.2. Was effectiveness established through an overview of clinical studies?	✓ A systematic review and meta-analysis for survival and another systematic review for utilities. Authors reported undertaking a review to identify studies, though brief	✗Data sourced from two RCTs	✗ Single RCT	✗ Single RCT	✗ Single RCT	✗Single RCT	✗Single RCT	✗Single RCT	✗Single RCT	✗Single RCT
3.3. Were observational data or assumptions used to establish effectiveness? If so, what are the potential biases in results?	✗	✗	✗	✗	✗	✗	✗	✓Potential bias arises from participants assigning themselves to an intervention and rejecting randomisation (selection bias)	✗	✗
**4. Were all the important and relevant costs and consequences for each alternative identified?**	✓ Costs<br>✗ Outcomes as no measure of HRQoL or utility included	✓ Outcomes<br>✗ Costs	✓ Outcomes<br>✗ Costs	✓ Outcomes<br>✗ Costs	✓	✓ Outcomes<br>✗ Costs	✓	✓	✓	✓
4.1. Was the range wide enough for the research question at hand?	✓	✗ Authors state that the analysis is "partial societal" but only include healthcare system and patient costs. In addition, only healthcare costs related to CVD were included	✗Only intervention costs were included (other healthcare costs etc were excluded). This is a very narrow perspective	✗Costs were limited only to those related to cardiac events, which ignores a relationship between cardiac health and general health, which is limited	✓Although it is not clear whether life expectancy was taken into account	✗Costs were limited only to those related to cardiac events, which ignores a relationship between cardiac health and general health, which is limited	✓ Seems fine, although hard to judge without an explicit perspective	✓/✗Authors included a wide range of costs, however it could be improved with the inclusion of primary care costs	✓	✓

Supplementary material

4.2. Did it cover all relevant viewpoints? (Possible viewpoints include the community or social viewpoint, and those of patients and third-party payers. Other viewpoints may also be relevant depending upon the particular analysis.)	✓ For the stated perspective, though the exclusion of primary healthcare costs may underestimate total costs	✗See above comments	✗See above comments	✗The authors stated a societal perspective however it appeared to be more of a healthcare payer perspective and was limited to cardiac related costs	✓ Fit the stated perspective, however, like the rest of the studies it could have been expanded (e.g.to include indirect costs)	✗The evaluation was limited to cardiac related costs	✓ Fine for a healthcare payer perspective, however, like the rest of the studies it could have been expanded (e.g.to include indirect costs)	✓	✓ Fine for a healthcare payer perspective, however, like the rest of the studies it could have been expanded (e.g.to include indirect costs)	✓
4.3. Were the capital costs, as well as operating costs, included?	✓	✓	✗	✓	✓	✓	✓	✓	✓	✓
**5. Were costs and consequences measured accurately in appropriate physical units (e.g. hours of nursing time, number of physician visits, lost work-days, gained life years)?**	✓	✓	✓	✓	✓	✓	✓	✓	✓	✓
5.1. Were any of the identified items omitted from measurement? If so, does this mean that they carried no weight in the subsequent analysis?	✗	✗	✓ Assuming that resource use was collected in the previous trial	✗However, it would have benefitted from including costs relating to cardiac health	**NR**	✗However, it would have benefitted from including costs relating to cardiac health	**NR**	✗However, it would have benefitted from the inclusion of primary care costs	✗	✗
5.2. Were there any special circumstances (e.g., joint use of resources) that made measurement difficult? Were these circumstances handled appropriately?	✗	✗	**NR**	**NR**	**NR**	✗	**NR**	✗	**NR**	**NR**
**6. Were the cost and consequences valued credibly?**	✓	✓/✗	✓	✓	✓	✓/✗	✓	✓/✗	✓/✗	✓
6.1. Were the sources of all values clearly identified? (Possible sources include market values, patient or client preferences and views, policy-makers' views and health professionals' judgements)	✓	✗Whilst the sources of resource use are listed to sources of associated costs are not	✓	✓ Although as a minor point the weights used to calculated EQ-5D values were not reported	✓ However the price year was not reported	✗ There was some lack of clarity around utility data, e.g. whether it was self-reported or with the help of team, and sample size	✓	✗The source for costing patients time was not reported (just noted that it was in line with German guidelines)	✗The source for costing was not reported (simply stated national sources)	✓
6.2. Were market values employed for changes involving resources gained or depleted?	✓	**NR**	✓	**NR**	**NR**	✓	**NR**	✓	**NR**	✓
6.3. Where market values were absent (e.g. volunteer labour), or market values did not reflect actual values (such as clinic space donated at a reduced rate), were adjustments made to approximate market values?	**NR**	**NR**	✓	**NR**	**NR**	**NR**	**NR**	✓	**NR**	✓
6.4. Was the valuation of consequences appropriate for the	✓	✓	✓	✓	✓	✓	✓	✓	✓	✓

Supplementary material

question posed (i.e. has the appropriate type or types of analysis – cost-effectiveness, cost-benefit, cost-utility – been selected)?										
**7. Were costs and consequences adjusted for differential timing?**	✓	✓	Not relevant	Not relevant	Not relevant	✗	Not relevant	Not relevant	Not relevant	Not relevant
7.1. Were costs and consequences that occur in the future 'discounted' to their present values?	✓	✓	Not relevant	Not relevant	Not relevant	✗	Not relevant	Not relevant	Not relevant	Not relevant
7.2. Was there any justification given for the discount rate used?	✓	✓	Not relevant	Not relevant	Not relevant	✗	Not relevant	Not relevant	Not relevant	Not relevant
**8. Was an incremental analysis of costs and consequences of alternatives performed?**	✓ The threshold for cost-effectiveness was also made explicit	✓ The threshold for cost-effectiveness was also made explicit	✓ The threshold for cost-effectiveness was also made explicit	✓ A threshold for cost-effectiveness was not provided but as the intervention dominated this was less relevant	✓ No threshold for cost-effectiveness was stated, though with the very high ICER this adds less value	✓The threshold for cost-effectiveness was also made explicit	✓Although a threshold for cost-effectiveness was not made explicit	✓ Although a threshold for cost-effectiveness was not made explicit	✓ The threshold for cost-effectiveness was also made explicit	✓The threshold for cost-effectiveness was also made explicit
8.1. Were the additional (incremental) costs generated by one alternative over another compared to the additional effects, benefits, or utilities generated?	✓	✓	✓	✓	✓	✓	✓	✓	✓	✓
**9. Was allowance made for uncertainty in the estimates of costs and consequences?**	✓Comprehensive (one-way sensitivity analysis, two-way sensitivity analysis and PSA)	✓Comprehensive (one-way sensitivity analysis and PSA)	✓PSA	✓PSA	✓Comprehensive (PSA and one-way scenario analysis)	✓Comprehensive (PSA and one-way scenario analysis)	✓Comprehensive (PSA and one-way scenario analysis)	✓Comprehensive (PSA and one-way scenario analysis)	✓PSA	✓Comprehensive (PSA and one-way scenario analysis)
9.1. If data on costs and consequences were stochastic (randomly determined sequence of observations), were appropriate statistical analyses performed?	✓	✓	✓	✓	✓	✓	✓	✓	✓	✓
9.2. If a sensitivity analysis was employed, was justification provided for the range of values (or for key study parameters)?	✗	✓	✓	✓	✓	✓	✓	✓	**NR**	✓
9.3. Were the study results sensitive to changes in the values (within the assumed range for sensitivity analysis, or within the confidence interval around the ratio of costs to consequences)?	✓	✓	✓	✓	✓	✓	✓	✓	**NR**	✓
**10. Did the presentation and discussion of study results include all issues of concern to users?**	✓/✗	✓/✗	✓/✗	✓/✗	✓/✗	✓/✗	✓	✓/✗	✓/✗	✓/✗
10.1. Were the conclusions of the analysis based on some overall index	✓	✓	✓	✓	✓	✓	✓	✓	✓	✓

Supplementary material

or ratio of costs to consequences (e.g. cost-effectiveness ratio)? If so, was the index interpreted intelligently or in a mechanistic fashion?										
10.2. Were the results compared with those of others who have investigated the same question? If so, were allowances made for potential differences in study methodology?	✓	✓	✓	✓	✗	✓	✓	✗	✓	✓
10.3. Did the study discuss the generalisability of the results to other settings and patient/client groups?	✗	✗	✗	✓	✓	✗	✓	✓	✗	✗
10.4. Did the study allude to, or take account of, other important factors in the choice or decision under consideration (e.g. distribution of costs and consequences, or relevant ethical issues)?	✓	✓	✓	✓	✓	✗	✓	✓	✓	✓
10.5. Did the study discuss issues of implementation, such as the feasibility of adopting the 'preferred' programme given existing financial or other constraints, and whether any freed resources could be redeployed to other worthwhile programmes?	✗	✗	✗	✗	✗	✗	✓	✗	✗	✗
